# The cell wall lipoprotein CD1687 acts as a DNA binding protein during deoxycholate-induced biofilm formation in *Clostridioides difficile*

**DOI:** 10.1038/s41522-023-00393-5

**Published:** 2023-05-11

**Authors:** Emile Auria, Lise Hunault, Patrick England, Marc Monot, Juliana Pipoli Da Fonseca, Mariette Matondo, Magalie Duchateau, Yannick D. N. Tremblay, Bruno Dupuy

**Affiliations:** 1 Institut Pasteur, Université Paris-Cité, UMR-CNRS 6047, Laboratoire Pathogenèse des Bactéries Anaérobies, F-75015 Paris, France; 2 Institut Pasteur, Université Paris-Cité, INSERM UMR1222, Unit of Antibodies in Therapy and Pathology, Paris, France; 3 Sorbonne Université, INSERM, CNRS, Centre d’Immunologie et des Maladies Infectieuses (CIMI-Paris), F-75013 Paris, France; 4 Plateforme de Biophysique Moléculaire, Institut Pasteur, CNRS UMR3528, Paris, France; 5 Plateforme Technologique Biomics, Institut Pasteur, Paris, France; 6 Plateforme Proteomic, Institut Pasteur, Paris, France; 7 Department of Biochemistry, Microbiology and Immunology, University of Saskatchewan, Saskatoon, SK Canada

**Keywords:** Cellular microbiology, Biofilms, Microbial genetics

## Abstract

The ability of bacterial pathogens to establish recurrent and persistent infections is frequently associated with their ability to form biofilms. *Clostridioides difficile* infections have a high rate of recurrence and relapses and it is hypothesized that biofilms are involved in its pathogenicity and persistence. Biofilm formation by *C. difficile* is still poorly understood. It has been shown that specific molecules such as deoxycholate (DCA) or metronidazole induce biofilm formation, but the mechanisms involved remain elusive. In this study, we describe the role of the *C. difficile* lipoprotein CD1687 during DCA-induced biofilm formation. We showed that the expression of *CD1687*, which is part of an operon within the *CD1685-CD1689* gene cluster, is controlled by multiple transcription starting sites and some are induced in response to DCA. Only CD1687 is required for biofilm formation and the overexpression of CD1687 is sufficient to induce biofilm formation. Using RNAseq analysis, we showed that CD1687 affects the expression of transporters and metabolic pathways and we identified several potential binding partners by pull-down assay, including transport-associated extracellular proteins. We then demonstrated that CD1687 is surface exposed in *C. difficile*, and that this localization is required for DCA-induced biofilm formation. Given this localization and the fact that *C. difficile* forms eDNA-rich biofilms, we confirmed that CD1687 binds DNA in a non-specific manner. We thus hypothesize that CD1687 is a component of the downstream response to DCA leading to biofilm formation by promoting interaction between the cells and the biofilm matrix by binding eDNA.

## Introduction

Gastrointestinal infections are a major public health issue. In high-income countries, the Gram-positive spore-forming anaerobe *Clostridioides difficile* is the leading cause of nosocomial diarrhea and colitis in adults receiving antibiotic treatments^1,2^. Moreover, *C. difficile* infections (CDI) can be persistent, which is a major challenge in the management of CDI following anti-*C. difficile* antibiotic treatment. Recurrent CDI occur in more than 20% of patients that receive antibiotics to treat their first CDI episode and this rate increases following new episodes^3,4^. The causes of recurrences have not been fully elucidated. Recurrence can be caused by either reinfection with a new strain or relapse with the same strain, suggesting that *C*. *difficile* can persist in the gastrointestinal tract^5^. Relapses were initially correlated with *C. difficile* ability to sporulate during the infection and resist antibiotic treatment^6,7^. However, relapses are also hypothesized to be associated with the persistence of *C. difficile* as a biofilm^8,9^. Persistent and chronic infections caused by different pathogens are known to be associated with biofilm formation^10^. It is estimated that at least 60% of all nosocomial and chronic bacterial infections are biofilm-associated^11^. In support of this hypothesis, *C. difficile* was recently showed to integrate biofilms formed by the colonic microbiota and this biofilm acted as a reservoir for persistence and recurrence in a laboratory model of CDI^9^.

Biofilms are structured communities of microorganisms associated with surfaces and encased in a self-produced extracellular matrix, which varies between bacterial species^12^. *C. difficile* can form biofilms as a single species or with other bacteria on various abiotic surfaces and several in vitro systems^9,13–15^. Moreover, *C. difficile* can integrate in vivo multi-species communities during a mouse infection, suggesting its ability to integrate mucosal biofilms^16^. In addition, *C. difficile* can form patchy glycan-rich biofilm-like structures in a mono-associated mouse model^17^. Although *C. difficile* can integrate multi-species biofilms in the gastrointestinal tract, there is limited knowledge on the biology of *C. difficile* biofilm formation in response to the gastrointestinal environment. During an infection, pathogens encounter several environmental factors including the presence of antibiotics, bile salts, osmotic pressure and varying nutrient sources and these are known to be important signals for biofilm formation during colonization^18,19^. Interestingly, *C. difficile* would face different challenges during dysbiosis as it changes the nutritional environment, bile salt metabolism, and osmotic and oxidative/nitrosative stresses^20^. Any of these factors could induce biofilm formation. For example, sub-inhibitory concentrations of antibiotics used to treat CDI enhance biofilm formation in vitro^21,22^. Furthermore, we recently demonstrated that sub-inhibitory concentrations of the secondary bile salt deoxycholate (DCA) enhances *C. difficile* biofilm formation^15^. In the DCA-induced biofilm, vegetative cells are protected from the toxicity of DCA as well as antibiotics and antimicrobial peptides^15^. We showed that biofilms induced by DCA are formed due to metabolic adaptation and reprogramming that are dependent on the available nutrients and excreted metabolites. Overall, excreted pyruvate is critical for the induction of biofilm formation^23^.

In addition to environmental factors inducing biofilm formation, several cellular factors, including cell surface components and regulators, have been shown to influence biofilm formation by *C. difficile*^24^. Among the genes that were upregulated in response to DCA, a gene encoding a lipoprotein (CD1687) is essential for biofilm formation in response to DCA^15^. The aim of this study was to characterize the role of CD1687 during biofilm formation by *C. difficile* in response to DCA. We demonstrated that CD1687 is exposed and active at the surface of the bacteria and that it binds DNA in vitro. This suggests that CD1687 acts as a protein anchoring the cells to the extracellular DNA (eDNA) present in the biofilm matrix.

## Results

### Genes of the *CD1685-CD1689* locus form an operon but multiple transcription start site control their expression

**Fig. 1:**
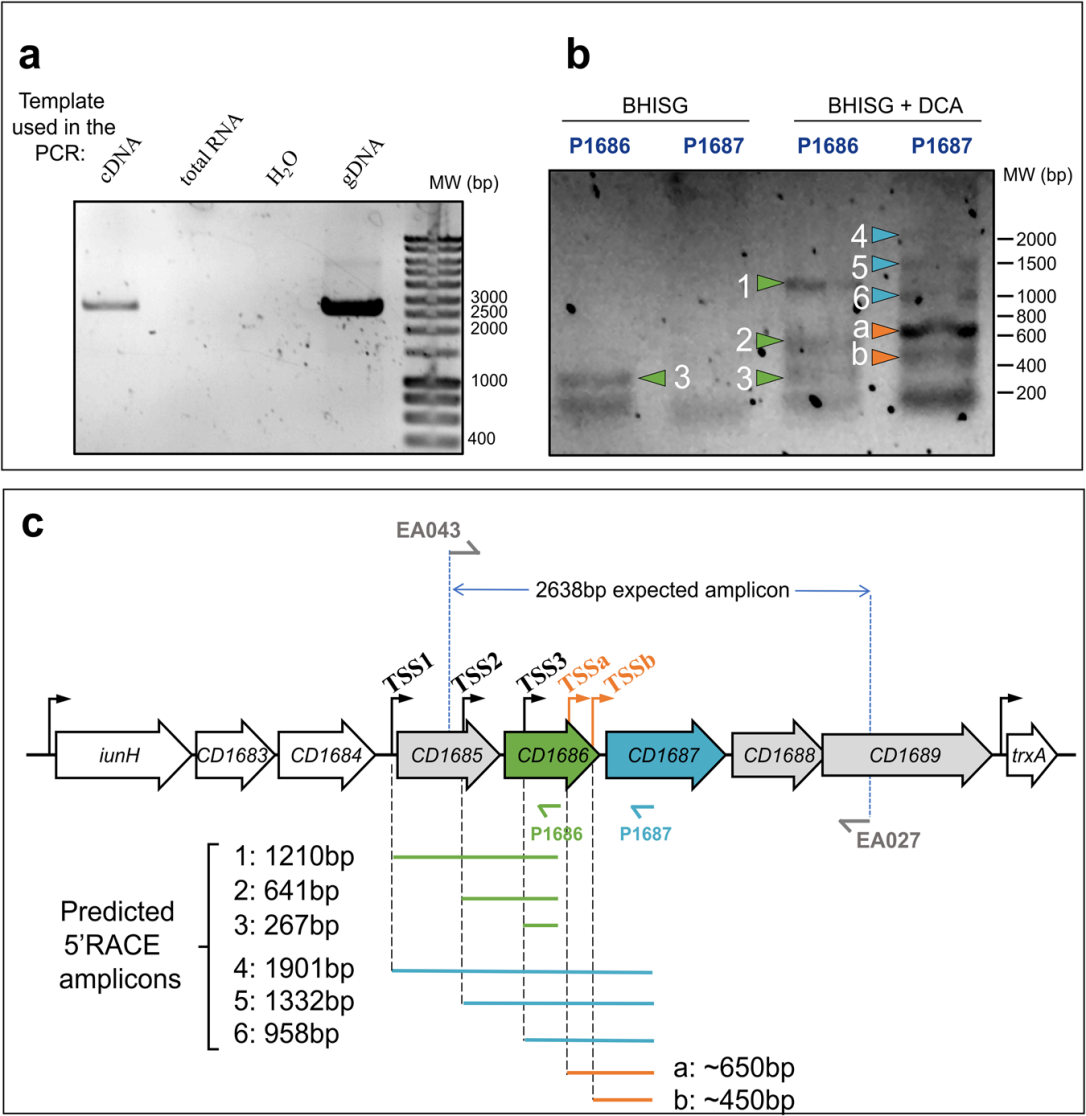
The *CD1685-CD1689* cluster in *C. difficile* strain 630Δ*erm* forms an operon with multiple transcription start sites. **a**.RT-PCR performed with primers EA043 and EA027 (Supplementary Table 1) from various nucleic acid templates. cDNA was obtained using the EA027 primer with total RNA extracted from 48 h biofilms grown in BHISG supplemented with DCA (240 µM). **b** 5’RACE results from amplification of the poly-guanylated cDNA obtained, respectively, with the EA021 and EA018 primers (Supplementary Table 1), then the P1686 or P1687 primers along with the universal amplification primer (AAP) from the 5’RACE kit. The RNA was extracted from 48 h cell cultures grown under biofilm-inducing conditions (BHISG + 240 µM DCA) or non-biofilm-inducing conditions (BHISG). **c** Organization of the *CD1685-CD1689* cluster, the location of the primers used for RT-PCR and the amplicons from the 5’RACE results using the P1686 or P1687 primers (amplicon sizes were predicted from the TSS identified by Soutourina et al. (2020) and Fuchs et al. (2021). TSS: Transcriptional Start Site; cDNA: complementary DNA; gDNA: genomic DNA. Blots in **a** and **b** derive from the same experiments and were not processed.

In previous transcriptomic experiments, we observed that the majority of genes in the *CD1685-CD1689* cluster were upregulated in the 48 h DCA-induced biofilm formed by *C. difficile* strain 630∆*erm*^15,23^. However, inactivation of CD1687 but not CD1688 prevented DCA-induced biofilm formation. To verify that the *CD1685-CD1689* genes formed an operon, RT-PCR experiments were performed with RNA extracted from cells grown under biofilm-inducing conditions (BHISG with 240 µM DCA). We observed a unique transcript spanning *CD1685* to *CD1689* suggesting the presence of at least one polycistronic mRNA at this locus (Fig. 1a). We then performed qRT-PCR to confirm that the five genes were upregulated at 48 h in the presence of DCA and only small difference in the fold changes were seen (Supplementary Figure 1a).

When looking at our previous RNAseq experiments, we observed a mapping bias of the sequencing reads favouring CD1687, CD1688, and CD1689 (Supplementary Figure 1b). Interestingly, recent analyses predicted three transcription starting sites (TSS) for the *CD1685-CD1689* locus: one upstream of the *CD1685* gene (TSS1), one upstream the *CD1686* gene (TSS2), and one in the coding sequence of *CD1686* (TSS3)^25,26^ (Fig. 1c). To confirm the existence of multiple TSS, 5’-RACE experiments were performed with total RNA extracted from cells grown for 48 h in BHISG with DCA (i.e., biofilm-inducing) or without DCA (i.e., non-biofilm inducing). The initial reverse transcriptions were performed with two primers annealing either the coding sequence of *CD1686* (P1686) or the coding sequence of *CD1687* (P1687) (Fig. 1b, c). In the absence of DCA, only one amplicon was observed, which is associated with the TSS inside *CD1686*. This amplicon was detectable when the P1686 primer was used but not with the P1687 primer. In the presence of DCA, we observed amplicons corresponding to the three predicted TSS with either primer (P1686 or P1687) and two additional amplicons were detected with P1687. This suggests that these two additional TSS (TSSa and TSSb; Fig. 1c) are active in the presence of DCA and one of these (TSSa) appears to be the most active of all TSS (Fig. 1b). Each amplicon was sequenced (Supplementary Table 2) and the location of TSS1, TSS2, and TSS3 closely matched their predicted location. However, high variation of the sequences for TSSa and TSSb made it difficult to identify their exact location. Overall, the transcription of the *CD1685-CD1689* operon is initiated from multiple TSS in the presence of DCA, suggesting that multiple factors are integrated to regulate the expression of the *CD1685-1689* operon to reflect the state of the bacterial population.

### Overexpressing CD1687 induces biofilm formation in the absence of DCA

We previously inactivated *CD1687* using the Clostron system^15^ but this approach is known to have some limitations. To confirm that only *CD1687* was required for biofilm formation, deletion of *CD1686*, *CD1687,* and *CD1688-CD1689* were generated (Supplementary Figure 2a). As observed before, only the deletion of *CD1687* negatively affected biofilm formation and complementation restore the phenotype (Supplementary Figure 2bc). Interestingly, deletion of *CD1686* removed TSS3, TSSa and TSSb suggesting that TSS1 and/or TSS2 are sufficient for the transcription of CD1687 in the presence of DCA resulting in biofilm formation.

Since CD1687 is required for DCA-induced biofilm formation and previously localized in the cell wall fraction^15^, we hypothesized that CD1687 is a DCA-sensing protein. To test this hypothesis, we verified the ability of CD1687 to directly interact with DCA using surface plasmon resonance. We showed that CD1687 can interact with DCA (Supplementary Figure 3). However, the dissociation constant is high (Kd of 1.65 ± 0.58 mM), and the estimated stoichiometry of the interaction is 5 ± 1 DCA molecules for one CD1687 protein, which implies that the interaction is not specific.

**Fig. 2:**
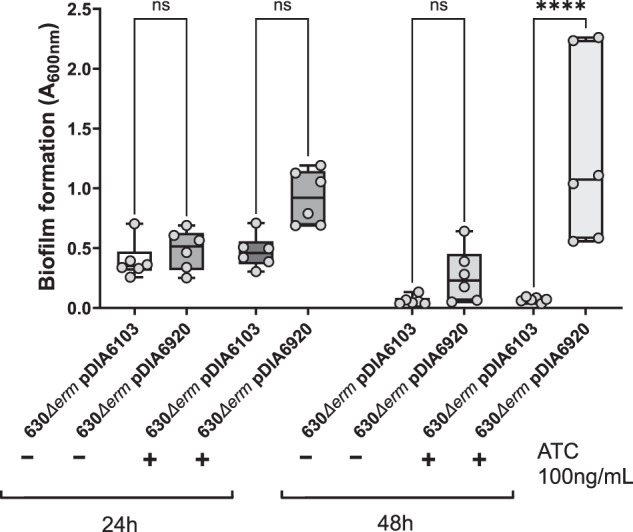
Overexpression of CD1687 induces biofilm formation in the absence of DCA. Biofilms formation was assayed 24 h or 48 h after inoculation in BHISG +/− ATC (100 ng/mL) with the wild-type strain (630Δ*erm*) containing either a control empty vector (pDIA6103) or the vector allowing the expression of CD1687 under the inducible *P*_*tet*_ promoter (pDIA6920). Each data point represents an independent biological replicate composed of 2 to 4 technical replicates. The boxplot used to represent quantitative data figure the median, minimum, maximum, and upper and lower quartiles. Asterisks indicate statistical significance with a one-way ANOVA test followed by a Tukey’s multiple comparison test (ns: not significant; ****p < 0.0001).

Interestingly, we observed an increase in biofilm formation in the presence and, to a certain extent, in the absence of DCA when the ∆*1687* mutant was complemented with an inducible plasmid-borne CD1687 (pDIA6920) (Supplementary Figure 2C). Although the increase was not significant, it suggested that CD1687 could induce biofilm formation in the absence of DCA. To test this hypothesis, pDIA6920 was introduced in the wild-type strain and its ability to form biofilm in the absence of DCA was evaluated with and without the addition of the inducer ATC. When CD1687 was overexpressed, a stronger biofilm was detectable at 24 h and 48 h (Fig. 2). Taken together, our results suggest that CD1687 expression is critical for biofilm formation which does not require DCA for its activity.

### CD1687 affects the expression of several transporter and metabolic priorities

As CD1687 is essential for DCA-induced biofilm formation and its overexpression can induce biofilm formation in the absence of DCA, we sought to identify genes whose expression is modified in the presence of CD1687 during the biofilm formation process. To do so, we performed two transcriptomic analyses: one comparing the wild type and the *∆1687* mutant grown in presence of DCA for 24 h, and the second comparing the wild type containing either the CD1687 inducible plasmid (pDIA6920) or an empty vector, both grown in the absence of DCA and in the presence of ATC as an inducer for 24 h.

**Fig. 3:**
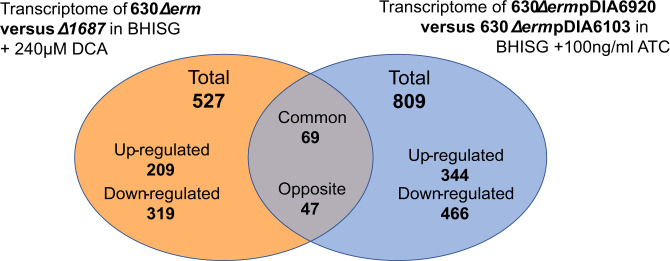
Differences in gene expression in the two transcriptomics experiments. Venn diagram of the genes differentially regulated in the two transcriptomics experiments performed in this study (Supplementary Table 4).

A total of 527 genes had a significant differential expression with a fold change <0.5 or >2 in the wild-type strain compared to the *∆1687* mutant under biofilm-inducing conditions (+DCA) (Fig. 3). In the presence of DCA, CD1687 seems to mainly downregulate the cell wall reticulation (*vanY2Y3*) as well as several uncharacterized regulators (Supplementary Figure 4, Supplementary Table 3). There seems to be a shift in membrane transporters that may result in an increase in the importation of branched-chain amino acids, iron, and a change in sugar transport (Supplementary Table 3). In terms of metabolism, the cells shift from the utilization of succinate (*CD2338-CD2344*), the Wood-Ljungdahl pathway, and the biosynthesis of aromatic amino acids to the fermentation of acetoin, leucine, branched chain amino acids and glycine (Supplementary Figure 4, Supplementary Table 3).

When CD1687 was overexpressed, 809 genes were differentially expressed, 343 genes were upregulated and 466 were downregulated (Fig. 3). As described in Supplementary Figure 4, changes in gene expression indicate a shift in transporters, metabolism, and regulation. Specifically, the expression of several sugar transporters is increased whereas the expression of the branched chain amino acids, methionine, alanine, and glycine transporters is downregulated (Supplementary Table 3). In terms of metabolism, genes involved in acetoin utilization, Stickland fermentations involving aromatic amino acids or leucine, the Wood-Ljungdahl pathway, and the pentose phosphate pathway are upregulated as well as those involved in the biosynthesis of several amino acids such as histidine, isoleucine, valine, and cysteine (Supplementary Table 3). The *dltABCD* operon is upregulated suggesting an increase of the D-alanylation of the teichoic acids (*dltABCD*). Interestingly, we noted that the gene cluster encoding the flagellum and genes associated with sporulation were upregulated.

When we compared both transcriptomic analyses, few genes overlapped between both analyses. Only 69 genes changed in the same direction whereas 47 genes were regulated in opposite direction (Fig. 3). The remaining 1220 genes were differentially expressed only under either condition (Fig. 3). The genes that were regulated in both conditions include those involved in cysteine synthesis (*cysE*, *cysK*), leucine utilization in Stickland fermentation (*hadABCI*), acetoin fermentation (*acoABCL*), cell wall proteins (*cwp9*, *cwp12*), some transporters (*alsT* transporting alanine or glycine, *rbsK* transporting ribose) and regulation (*sinRR’*). Overall, this suggests that CD1687 induces metabolic re-organization, including those occurring in response to DCA that leads to biofilm formation^23^.

However, these changes do not fully align with our previous analyses^23^. We previously observed that DCA causes the up-regulation of gene involved in butanoate, lactate, and acetate fermentations, a shift in Stickland fermentations from the use of aromatic amino acids to the use of branched chain amino acids and glycine, and the down-regulation of genes involved in glycolysis, glucose intake, and sporulation^23^. These changes were not observed when CD1687 was overexpressed suggesting that CD1687 is not involved in those processes or does not mediate the immediate response to DCA. CD1687 is probably part of the downstream response and may interact with other proteins to promote these changes.

### CD1687 interacts with several cell wall proteins

Given that CD1687 is a cell wall protein^15^ that does not have a transmembrane domain but probably anchored to the cell surface membrane via a myristoyl anchor^27^, we hypothesized that CD1687 induces transcriptional changes by transmitting external signals by interacting with membrane proteins. To find these potential proteins, we performed a pull-down assay using crude extracts of *C. difficile* cells overexpressing a C-terminal hexahistidine-tagged CD1687 in BHISG without DCA (Supplementary Table 5). They were compared to control extracts collected from a *C. difficile* mutant ∆*1687* with the empty vector in the same conditions. Among the 43 proteins identified only in the test samples and not in the control samples, which included the CD1687 protein (Supplementary Table 5), four are predicted to be membrane proteins and include a component of sugar transporter (CD2667) and a sodium symporter (CD2693). We also identified four proteins that belong to the large family of solute-binding proteins associated with ABC transporters and one nucleotide phosphodiesterase (CD0689). These five proteins could be involved in signal transport and cellular response leading adaptation in different environmental conditions^28,29^. Among the membrane proteins, we also found a putative lipoprotein (CD0747) and a LCP (LytR-CpsA-Psr) family protein (CD2766) involved in the cell wall polysaccharide assembly^30^. We noted that only one encoding gene of protein partners (CD0037) was upregulated in both transcriptomes (Supplementary Table 5), which is typically localized in the cytoplasm. Since most of the membrane proteins identified by the pull-down experiment are cell wall proteins involved in membrane transport, it is possible that CD1687 directly affects transport of different nutrients and is consistent with the observed effect in our transcriptomes.

### CD1687 is exposed at the cell surface

**Fig. 4:**
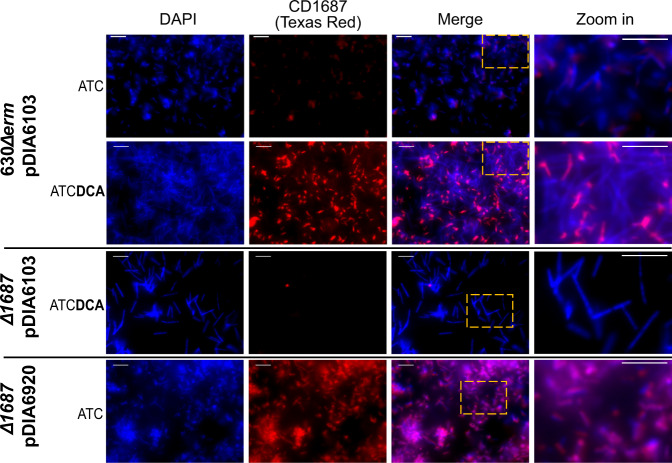
CD1687 localizes at the cell surface of *C. difficile* and displays heterogenous distribution within the biofilm. In situ epifluorescence microscopy analysis was performed on 48 h biofilms grown in BHISG + ATC (100 ng/mL) either in the presence or absence of DCA (240 µM) as indicated. The strains tested were the wild-type strain (630Δ*erm*) carrying the control vector pDIA6103 and with the ∆*1687* strain carrying the plasmid with an inducible CD1687 (pDIA6920) or the control plasmid (pDIA6103). DNA is stained with DAPI (blue) and CD1687 is labeled with specific anti-CD1687 rabbit antibodies detected with a TexasRed-conjugated goat anti-rabbit antibody (red). Pictures are representative of three biological replicates and were taken with a Nikon Eclipse Ti inverted microscope (Nikon, Japan). Scale bar: 10 µm.

Since CD1687 was detected in the cell wall fraction^15^, we wondered whether CD1687 is exposed at the cell surface. To verify this, we performed epifluorescence microscopy analysis of *C. difficile* 630Δ*erm* strain and its derivatives using rabbit polyclonal antibodies raised against CD1687. When grown 48 h in BHISG with or without DCA, no signal was observed in the ∆*1687* mutant confirming the specificity of our antibody (Fig. 4 and Supplementary Figure 5). For the wild-type strain, we observed a weak signal when grown in absence of DCA, confirming that this protein is expressed at low levels under non-biofilm-inducing conditions. In the presence of DCA, the signal was stronger in the presence of DCA, although the expression of CD1687 was not homogeneous in the population. In contrast, the signal for CD1687 is homogeneous in the population of the complemented ∆*1687* strain (Fig. 4 and Supplementary Figure 5). Since the cells were not permeabilized during the experiment and PFA does not significantly affect membrane permability^31^, we concluded that CD1687 is exported to the cell wall and exposed at the cell surface.

**Fig. 5:**
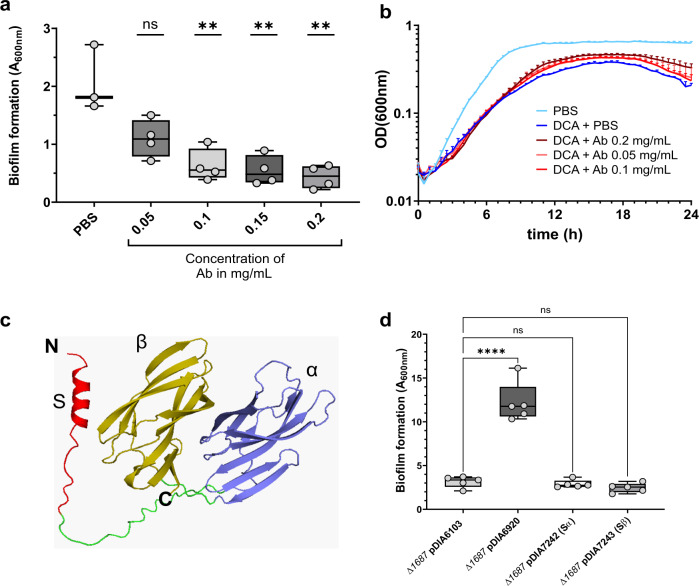
DCA-induced biofilm formation is inhibited in the presence of anti-CD1687 antibodies. **a** Biofilm formation of the 630*Δerm* strain was assayed 48 h in BHISG with DCA (240 µM) cultures in presence of different concentration of anti-CD1687 rabbit antibodies (0.05 mg/mL to 0.2 mg/mL). **b** Growth kinetics (OD_600nm_) of the WT (630*Δerm*) in BHISG medium with PBS or DCA supplemented with different concentrations of anti-CD1687 rabbit antibodies (0.05 mg/mL to 0.2 mg/mL). Ab: antibody; nsAb: non-specific antibody. **c** The alphafold2 predicted structure of CD1687 show a N-terminal signal peptide S (red) connected to the α beta domain (purple) by a linker peptide (green), with another similar β beta domain (yellow) in the C-terminal region. **d** 48 h biofilms form by various *Δ1687* strain complemented with an empty vector (pDIA6103) or plasmids overexpressing the full-length CD1687 (pDIA6920) or truncated CD1687 lacking either one of the two domains removed (pDIA7242 and pDIA7243, Supplementary Table 1) grown in BHISG with ATC (100 ng/mL) and DCA (240 µM). Each data point represents an independent biological replicate composed of 2 to 4 technical replicates. The boxplots used to represent quantitative data figure the median, minimum, maximum, and upper and lower quartiles. Asterisks indicate statistical significance with a one-way ANOVA test followed by Dunnett’s multiple comparison test (**a**) (ns: not significant; **p < 0.01) or a Tukey’s multiple comparison test (**d**) (ns: not significant; ****p < 0.001).

Based on the cellular localization of CD1687, we wondered if the addition of the anti-CD1687 antibodies during growth could prevent DCA-induced biofilm formation. As shown in Fig. 5a, the addition of the anti-CD1687 polyclonal antibodies to cells grown under biofilm inducing conditions (BHISG + 240 µM DCA) strongly inhibited biofilm formation in a dose-dependent manner. No inhibitory effect was observed when an unpublished non-specific antibody was used at the highest concentration of anti-CD1687 that inhibited biofilm formation (data not shown). In addition, bacterial growth was unaffected by the antibodies, regardless of the concentration used in the biofilm assays (Fig. 5b). Therefore, inhibiting extracellular function of CD1687 prevents biofilm formation, indicating both that CD1687 is exposed at the cell surface and that its presence at the surface of the cell wall is critical for DCA-induced biofilm formation.

To get some insights on the structure-function of CD1687, we used the software AlphaFold2^32^ to predict the 3D protein structure of CD1687. As shown in Fig. 5c, CD1687 has an alpha helix N-terminal signal peptide and two putative beta domains. To search for possible functions of the beta domains, the putative structure of CD1687 was analyzed in the Ekhidna database through the Dali server^33^, but no function was detected. Since the function of CD1687 could be assigned to one of the two beta domains, we complemented the *∆1687* mutant by overexpressing CD1687 with either one of the two domains removed and growing these strains under biofilm-inducing conditions (BHISG + 240 µM DCA. Complementation of the mutant was not observed, indicating that *C. difficile* needs both beta domains of the CD1687 to form DCA-induced biofilms (Fig. 5d).

### CD1687 binds to DNA in a non-specific manner

**Fig. 6:**
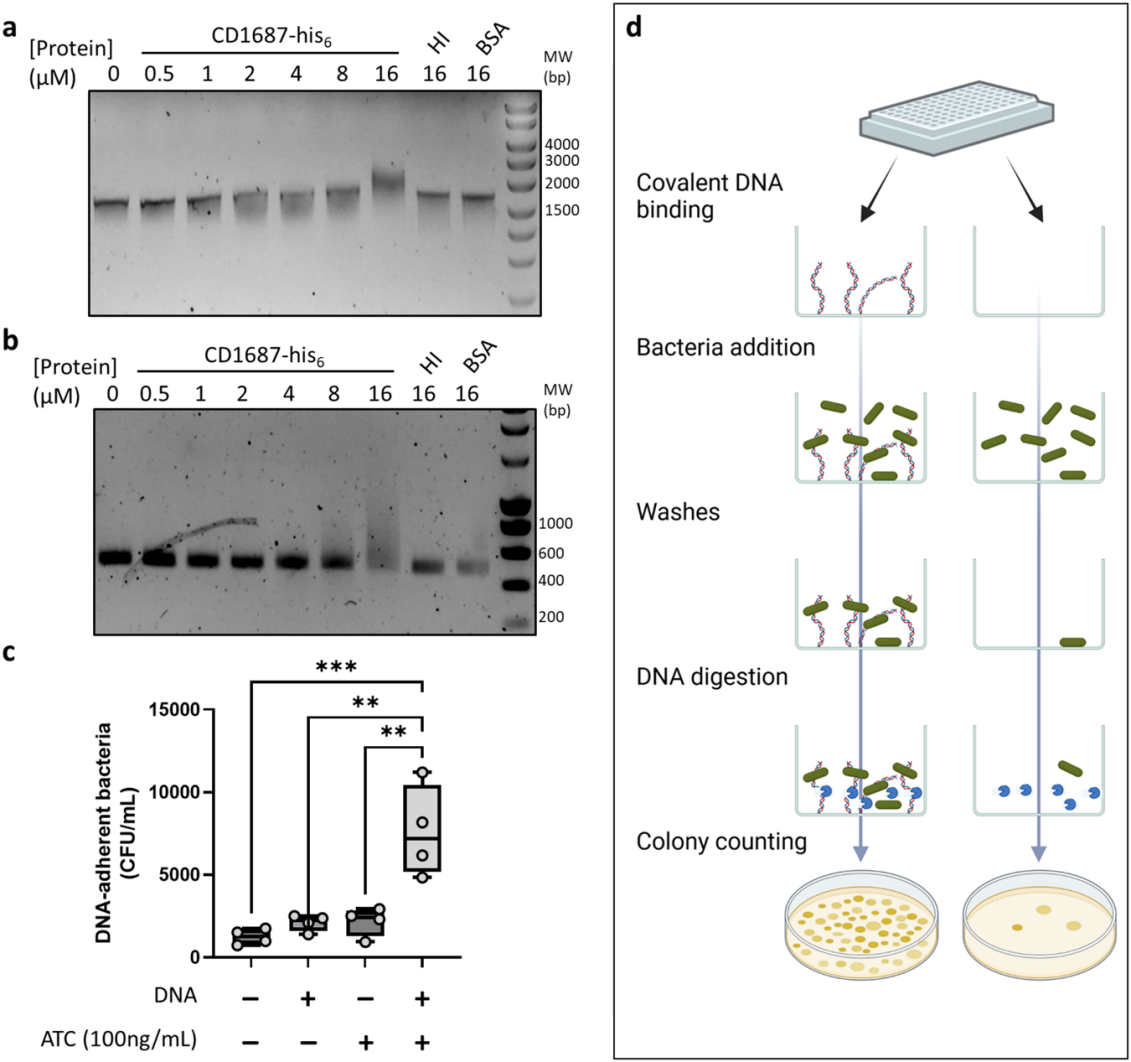
CD1687 binds DNA and shifts DNA migration. Electrophoretic Mobility shift assay (EMSA) was performed with **a** *E. coli* plasmid pUC9 or **b** *C. difficile* DNA (450 bp PCR-amplicon) mixed with various concentrations of CD1687 (up to 16 µM), with 16 µM of heat-inactivated (HI) CD1687 or BSA used as controls. **c** *C. difficile* CFUs measured from the adhesion assay. The *Δ1687 pDIA6920* strain was used expressing or not *CD1687* in response to ATc as described in (**d**). Schema of the adhesion assay. We compared the adhesion of bacteria either expressing CD1687 or not in wells that contain or not covalently bound DNA. This schema was made with biorender.com. The boxplot used to represent quantitative data figure the median, minimum, maximum, and upper and lower quartiles. Each data point represents an independent biological replicate. Asterisks indicate statistical significance with a one-way ANOVA test followed by Dunnett’s multiple comparison test (**p < 0.01; ***p < 0.001). Blots in **a** and **b** derive from the same experiments and were not processed.

Since we did not identify a potential function from the CD1687 structure, we sought to determine if CD1687 has a DNA-binding activity as observed for *Staphylococcus aureus* lipoproteins that promote eDNA-dependent biofilm formation^34^. Since the *C. difficile* biofilm matrix is mainly composed of eDNA^15^, we tested the ability of CD1687 to bind to DNA by performing an electromobility shift assay (EMSA). When the purified CD1687 protein was incubated with the *E. coli* DNA plasmid pUC9 or a PCR-generated amplicon produced from *C. difficile* DNA (from a sequence in the region of *CD1438*), we observed that the migration of the DNA was shifted by the presence of the CD1687 and increasing CD1687 concentration correlates with more retention (Fig. 6a, b). However, we did not observe a shift when CD1687 was heat-inactivated or if BSA was used as control at the highest concentration of CD1687 that shift DNA fragments. To test whether CD1687 allows the anchoring of the bacteria to eDNA, we performed a DNA-binding experiment using whole *C. difficile* bacteria (Fig. 6d). We covalently linked the same amplicon used in EMSA (Fig. 6b) to a microarray plate before adding the *∆1687 pDIA6920* strain (Supplementary Table 1) producing or not CD1687. We then counted the bacteria linked to DNA after adding DNAse I in the wells (Fig. 6d). We found that bacteria adhered more to DNA in the wells when CD1687 was produced than when DNA or CD1687 was absent (Fig. 6c). Therefore, with this experiment and the EMSA results, we conclude that CD1687 can bind to eDNA in a non-specific manner. This binding activity likely allows *C. difficile* to anchor itself to eDNA in the biofilm.

## Discussion

In this study, we confirmed that only *CD1687* in the *CD1685-CD1689* cluster was required for DCA-induced biofilm formation and this required the localization of CD1687 at the cell surface.

In fact, this protein is detected in the cell wall^15^ as well as on the cell surface (Figs. 4 and 5a) yet it has been detected and described as a membrane-anchored lipoprotein^27^. The small size of the protein and its predicted structure imply that this protein should be closer to the membrane than to the surface of the cell wall. Since CD1687 can be easily recovered from the cell wall fraction, this suggests that there are possible as yet undescribed post-translational modifications on CD1687 that would cleave its myristoyl anchor, allowing the protein to bind to the cell wall. We noted that there is a significant heterogeneity in response to DCA for the expression and localization of CD1687 at cell surface in the population as observed by microscopy (Fig. 4 and Supplementary Figure 5). This would explain the relatively low transcriptional level of the *CD1685-CD1689* gene cluster at the population level^15^. Interestingly, the more CD1687 is homogeneously expressed in the cell population, the greater the biofilm formed (Fig. 4, Supplementary Figure 2c). To our knowledge, expression heterogeneity of critical biofilm components has not yet been reported in *C. difficile*. Phenotypic heterogeneity in biofilms is well characterized in several other bacterial species resulting in phenotypic diversification and division of labor in a clonal bacterial population^35^. For example, a subpopulation of cells synthesize the exopolysaccharides matrix during biofilm formation in *B. subtilis*^36^. Phenotypic heterogeneity has been described in planktonic cells of *C. difficile* and this affected the expression of the flagellum and toxins^37^. In this case, heterogeneity is controlled by a specific DNA recombination event mediated by RecV^38^ and the Rho factor^39^. In addition, *C. difficile* colony morphology is also subjected to phenotypic heterogeneity resulting in changes in bacterial physiology and pathogenesis and this occurs through phase variation of the CmrRST signal transduction system expression^40,41^.

Given that CD1687 forms an operon with a two-component regulatory system (CD1688-1689) and that CD1687 is a cell wall protein, we first hypothesized that CD1687 was involved in signal transduction leading to transcriptional modifications in response of DCA. However, CD1687 did not bind DCA, which eliminates the putative role of CD1687 as a DCA-sensing protein. Furthermore, with the exception of sporulation, genes regulated by CD1688^42^ have limited overlap suggesting that CD1687 may not be part of the CD1688-CD1689 signaling cascade. This is consistent with the absence of CD1689 and CD1688 in our pull-down assay. However, several solute-binding proteins and transporter-associated proteins were isolated in a pull-down assay. This and the transcriptional analysis provide evidence that CD1687 influences the metabolism of *C. difficile*. In support of this, regulators (Spo0A, CodY, and SinRR’) that manage metabolic priorities during growth phases, were differentially regulated when CD1687 was overexpressed^43–45^. Furthermore, the expression of the gene encoding toxin and those involved in sporulation were also affected and these processes are known to be dependent on the metabolic state of *C. difficile*. When we compared the genes differentially regulated in the absence of CD1687 under DCA-inducing conditions to those differentially regulated when CD1687 was overexpressed in the absence of DCA, there were only 69 common genes, which included genes involved in different metabolic pathways and transport. However, these changes in metabolism-associated genes did not overlap with our previous analyses on gene expression during DCA-induced biofilm formation^23^, suggesting that CD1687 is not part of the immediate response to DCA and probably plays a role in the downstream response. Taken together, our data suggest that CD1687 helps reorganize metabolic priorities in response to DCA but this hypothesis alone does not explain the role of CD1687 in the biofilm formation without DCA. Therefore, CD1687 may have additional roles.

Interestingly, many proteins found at the bacterial cell surface interact with eDNA found in the biofilm matrix and this contributes to the organization and structural stability of the biofilm^46^. Membrane lipoproteins have already been shown to directly interact with eDNA and participate in biofilm architecture. In *S. aureus*, several membrane-attached lipoproteins interacting with the eDNA of the biofilm matrix have been identified as promoting *S. aureus* biofilm formation^34^. Here we confirmed that CD1687 interacts in vitro with DNA in a non-specific manner both with the purified protein and the bacteria producing CD1687, whose level of production is sufficient to increase bacterial adhesion to eDNA. These results support the hypothesis that CD1687 acts as an eDNA-binding protein during biofilm formation by creating anchor points for eDNA on the cell surface. Similar to our observation with CD1687, overexpressing eDNA-binding proteins in *S. aureus* resulted in an increased retention of surface eDNA and an enhanced biofilm biomass. However, deleting the *S. aureus* lipoproteins had minimal impact on biofilm formation but biofilm porosity increase indicating that interactions of the lipoprotein with eDNA contribute to overall biofilm structure. Unlike the lipoprotein found in *S. aureus*, a deletion or inactivation of *CD1687* abolished biofilm formation^34^. CD1687 interacting with eDNA seems to be an essential part of DCA-induced biofilm formation. Other structures may also interact with eDNA. Recently, two minor subunits (PilW and PilJ) of the *C. difficile* T4P were shown to directly interact with eDNA to promote biofilm formation^47^. Neither subunit have a predicted DNA-binding motif as observed with CD1687. The T4P is a structure that promotes biofilm formation in the absence^48,49^ or presence of DCA^23^. In the presence of DCA, PilW is upregulated but is not required for biofilm formation^15,23^. Furthermore, the *pilW* gene was differently regulated in our transcriptome; upregulated in the WT vs *Δ1687* with DCA analysis (significantly but below the threshold) and downregulated in the overexpressed CD1687 vs WT without DCA analysis. Therefore, CD1687 and the T4P may have complementary role and the lack of eDNA-binding by one of these components may change the behavior of *C. difficile* during biofilm formation.

Despite the potential role of CD1687 as an eDNA-binding protein and in metabolism, we cannot exclude that the overexpression of CD1687 modifies the properties of the cell wall through the interactions of CD1687 with other membrane proteins and transporters (Supplementary Table 5). These interactions could be detected by different sensors, which would activate a feedback loop to modify the cell wall and the composition of the cell surface proteins. For example, the *dltABCD* operon was upregulated when CD1687 was overexpressed in the absence of DCA. The DltABCD proteins are responsible for the D-alanylation of teichoic acids, which changes the electrical charges of the cell wall and surface^50^. Overexpression of CD1687 also affected cell morphology; in response to DCA, cells expressing high levels of CD1687 show reduced size and shape distortion (Fig. 4 and Supplementary Figure 5). Overall, the overexpression of CD1687 may have downstream effects on the physiology of *C. difficile* and these changes may contribute to biofilm formation.

Finally, our hypothesis is that the mechanism for biofilm formation in the presence of DCA is different than the mechanism when DCA is absent and CD1687 is overexpressed. In the presence of DCA, we know that *C. difficile* goes through a metabolic re-organization^23^ and, based on our data, CD1687 would help with metabolic priorities for long-term adaptation. Once there is enough eDNA, CD1687 would interact with eDNA binding and serve as an anchor point. When CD1687 is overexpressed independently of DCA, it increases homogeneity of CD1687 surface localization in the population and serves as multiple anchoring sites for eDNA resulting in a strongly adherent biofilm. As observed in *S. aureus*, other lipoproteins may bind eDNA in *C. difficile* and several are upregulated in response to DCA^23^. Unlike the lipoproteins characterized in *S. aureus*, the lipoprotein CD1687 probably has a critical function in metabolism in response to DCA and other lipoproteins do not provide functional redundancy. This highlights the importance of CD1687 in promoting biofilm formation. More research will be needed to understand the role and the contribution of these other lipoproteins to biofilm.

## Methods

### Bacterial strains and culture conditions

Bacterial strains and plasmids used in this study are listed in Supplementary Table 1*C. difficile* strains were grown anaerobically (5% H2, 5% CO_2_, 90% N_2_) in TY medium (30 g/L tryptone, 20 g/L yeast extract) or in BHISG medium (BHI with 0.5% (w/v) yeast extract, 0.01 mg/mL cysteine and 100 mM glucose) and supplemented with cefoxitin (250 μg/ml), D-cycloserine (8 μg/ml) and thiamphenicol (15 μg/ml) when necessary. In addition, 100 ng/mL of anhydrotetracycline (ATC) was added to induce the *P*_*tet*_ promoter of pRPF185 vector derivatives in *C. difficile*. *E. coli* strains were grown in LB broth supplemented with chloramphenicol (15 µg/mL) and ampicillin (100 µg/mL).

### Biofilm assays

Overnight cultures of *C. difficile* grown in TY medium with appropriate antibiotics were diluted to 1/100 into fresh BHISG containing the desired supplements (240 µM DOC, 100 ng/mL ATC or both). Depending on the assay, the diluted cultures were then aliquoted either with 1 mL per well in 24-well plates (polystyrene tissue culture-treated plates, Costar, USA) or with 200 µL in 96-well plates (polystyrene black tissue-culture-treated plates, Greiner Bio One, Austria). The plates were incubated at 37 °C in an anaerobic environment for 48 h. Biofilm biomass was measured in the 24-well plates using an established method^15^. For biofilm assays in 96-well plates used for microscopy, spent medium was carefully removed by pipetting and 200 µL PBS supplemented with 4% of paraformaldehyde (PFA) were added. Plates were incubated for an hour at room temperature and the media was then carefully removed by pipetting before adding PBS for 48 h at 4 °C. In all assays, sterile medium was used as a negative control and a blank for the assays.

### Gene deletion in *C. difficile*

Gene deletion in *C. difficile* was performed as described in Peltier et al.^51^. Regions upstream and downstream of the genes of interest were PCR-amplified using primer pairs described in Supplementary Table 1. PCR fragments and linearized pDIA6754^51^ were mixed and assembled using Gibson Assembly (NEB, France) and transformed by heat shock in *E. coli* NEB 10β strain. The plasmid constructions were verified by sequencing and plasmids with the right sequences were transformed in *E. coli* HB101 (RP4). The resulting strains were used as donors in a conjugation assay with the relevant *C. difficile* strains. Deletion mutants were then obtained using a counter-selection as described in Peltier et al.^51^.

### Protein extraction from *C. difficile* and pull-down assay

*C. difficile* strains were anaerobically grown for 48 h in 20 mL BHISG cultures with ATC in tubes. Cells and biofilms were harvested by centrifugation (10 min; 14,000 × *g*; 4 °C) and washed in a cold phosphate buffer (50 mM; pH = 7.0; 4 °C). Cells were then resuspended in 1 ml of the same phosphate buffer containing the purified catalytic domain of the endolysin CD27L (3 µg/mL) and suspension was incubated 1 h at 37 °C to lyse the bacterial cells. The total extracts were then vortexed for 1 min and used for pull-down assay with Ni-NTA beads as described below for CD1687 purification from *E. coli* expression. Five biological replicates of each condition were used in the pull-down assay (Supplementary Table 5).

### Production and purification of CD1687 and anti-CD1687 antibodies

*E. coli* strain Bli5 containing a pET20-derived plasmid carrying the *CD1687* gene (Supplementary Table 1) was used to overexpress hexa-histidine-tagged CD1687 protein without its signal peptide. Cells were grown overnight at 37 °C in LB supplemented with glucose (1% w/v) and antibiotics (ampicillin 100 µg/mL and chloramphenicol 15 µg/mL). The overnight culture was transferred (1/100) in 1 L of the same medium and incubated at 37 °C. Once the culture reached an OD_600nm_ of 0.5, IPTG was added (final concentration 0.1 mM) and the culture was incubated for an additional 3 h. Cells were then harvested by centrifugation (5000 × *g*, 10 min, 4 °C) and the pellet was washed with cold PBS. After centrifugation, the supernatant was discarded and resulting pellet was frozen at −20 °C. The pellet was then resuspended in 15 mL of lysis buffer (50 mM sodium phosphate pH = 8.0; 300 mM NaCl) and sonicated. After centrifugation (5000 × *g*, 10 min, 4 °C), the supernatant was collected and mixed with Ni-NTA beads and incubated one hour at 4 °C. The beads were then transferred to an elution column and washed with washing buffer (50 mM sodium phosphate pH = 8.0; 300 mM NaCl; 10 mM imidazole). Proteins were eluted with 2 ml of sodium phosphate buffer (50 mM, pH = 8.0) supplemented with 300 mM NaCl and a gradient of imidazole ranging from 50 mM to 500 mM. Eluted proteins were analyzed by western immunoblotting and fractions containing CD1687 were dialyzed in TAE buffer (Tris-base (20 mM); acetic acid (10 mM); EDTA (0.5 mM); pH = 8.5) using Slide-A-Lyzer dialysis units (Thermo Fisher Scientific, USA). To raise polyclonal anti-CD1687 antibodies, two female rabbits (New Zealand White) were injected four times with 50 µg of purified CD1687(His_6_) (0.5 mL of antigen with 0.5 mL of complete Freund’s adjuvant at D0, D14, D28, and D42) with the Covalab company (France). Antibodies were purified at D53 of immunization.

### Real-time surface plasmon resonance binding assay

All experiments were performed on a Biacore T200 instrument (Cytiva, USA) equilibrated at 25 °C in buffer TAE (20 mM Tris base, acetic acid 10 mM, EDTA 0.5 mM, pH = 8.5). CD1687(His_6_) (100 µg/ml) was captured for 600 s at 2 µl/min on an NiCl_2_-loaded NTA sensorchip, reaching a surface density of 1000–1200 RU (resonance units; 1RU ≈ 1 pg/mm^2^). DCA (16–2000 µM) was then injected at 10 µl/min for 120 s, simultaneously on the CD1687 surface and on an empty reference chip from which non-specific signals were subtracted.

### Protein sequencing assay via mass spectrometry

#### Protein digestion

Proteins were reduced using 5 mM TCEP for 30 min at room temperature. Alkylation of the reduced disulfide bridges was performed using 10 mM iodoacetamide for 30 min at room temperature in the dark. Proteins were then digested in two steps, first with 250 ng r-LysC Mass Spec Grade (Promega) for 4 h at 30 °C then samples were diluted below 2 M urea with 100 mM Tris HCl pH 8.5 and 500 ng Sequencing Grade Modified Trypsin was added for the second digestion overnight at 37 °C. Proteolysis was stopped by adding formic acid (FA) at a final concentration of 5%. The resulting peptides were cleaned using AssayMAP C18 cartridges on the AssayMAP Bravo platform (Agilent) according to the manufacturer’s instructions. Peptides were concentrated to dryness and resuspended in 2% acetonitrile (ACN) and 0.1% FA just prior to LC-MS injection.

#### LC-MS/MS analysis

LC-MS/MS analysis was performed on a Q ExactiveTM Plus Mass Spectrometer (Thermo Fisher Scientific) coupled with a Proxeon EASY-nLC 1200 (Thermo Fisher Scientific). 500 ng of peptides were injected onto a home-made 37 cm C18 column (1.9 μm particles, 100 Å pore size, ReproSil-Pur Basic C18, Dr. Maisch GmbH, Ammerbuch-Entringen, Germany). Column equilibration and peptide loading were done at 900 bars in buffer A (0.1% FA). Peptides were separated with a multi-step gradient from 3 to 6% buffer B (80% ACN, 0.1% FA) in 5 min, 6 to 31% buffer B in 80 min, 31 to 62% buffer B in 20 min at a flow rate of 250 nL/min. Column temperature was set to 60 °C. MS data were acquired using Xcalibur software using a data-dependent method. MS scans were acquired at a resolution of 70,000 and MS/MS scans (fixed first mass 100 *m*/*z*) at a resolution of 17,500. The AGC target and maximum injection time for the survey scans and the MS/MS scans were set to 3E6, 20 ms and 1E6, 60 ms, respectively. An automatic selection of the 10 most intense precursor ions was activated (Top 10) with a 30 s dynamic exclusion. The isolation window was set to 1.6 *m*/*z* and normalized collision energy fixed to 27 for HCD fragmentation. We used an underfill ratio of 1.0% corresponding to an intensity threshold of 1.7E5. Unassigned precursor ion charge states as well as 1, 7, 8, and >8 charged states were rejected and peptide match was disable.

### Protein identification and quantification

Acquired Raw data were analyzed using MaxQuant software version 2.1.1.0^52^ using the Andromeda search engine^53,54^. The MS/MS spectra were searched against the *C.difficile* 630 database (3957 entries).

All searches were performed with oxidation of methionine and protein N-terminal acetylation as variable modifications and cysteine carbamidomethylation as fixed modification. Trypsin was selected as protease allowing for up to two missed cleavages. The minimum peptide length was set to 7 amino acids and the peptide mass was limited to a maximum of 4600 Da. The false discovery rate (FDR) for peptide and protein identification was set to 0.01. The main search peptide tolerance was set to 4.5 ppm and to 20 ppm for the MS/MS match tolerance. Second peptides were enabled to identify co-fragmentation events. A false discovery rate cut-off of 1% was applied at the peptide and protein levels. The mass spectrometry proteomics data have been deposited to the ProteomeXchange Consortium via the PRIDE partner repository with the dataset identifier PXD038282. The statistical analysis of the proteomics data was performed as described previously^55^. Briefly, four biological replicates were acquired per condition. To highlight significantly differentially abundant proteins between two conditions, differential analyses were conducted through the following data analysis pipeline: (1) deleting the reverse and potential contaminant proteins; (2) keeping only proteins with at least two quantified values in one of the two compared conditions to limit misidentifications and ensure a minimum of replicability; (3) log2-transformation of the remaining intensities of proteins; (4) normalizing the intensities by median centering within conditions thanks to the normalizeD function of the R package DAPAR^56^, (5) putting aside proteins without any value in one of both compared conditions: as they are quantitatively present in a condition and absent in another, they are considered as differentially abundant proteins and (6) performing statistical differential analysis on them by requiring a minimum fold-change of 2 between conditions and by using a LIMMA t test^57,58^ combined with an adaptive Benjamini–Hochberg correction of the *p* values thanks to the adjust.p function of the R package cp4p^59^. The robust method of Pounds and Cheng was used to estimate the proportion of true null hypotheses among the set of statistical tests^60^. The proteins associated with an adjusted *p* value inferior to an FDR level of 1% have been considered as significantly differentially abundant proteins. Finally, the proteins of interest are therefore the proteins that emerge from this statistical analysis supplemented by those being quantitatively absent from one condition and present in another.

### RNA isolation, qRT PCR

Cells were grown in 24-well plates and 10 wells per plate were used to produce one replicate for one condition. For biofilm conditions, the supernatant was removed by inverting the plate and the biofilms were carefully washed twice then resuspended in 3 mL of PBS. In other conditions, the whole bacterial population was collected and cells were harvested by centrifugation (10 min, 8000 × *g*, 4 °C) and resuspended in 1 ml of PBS. Cell suspensions in PBS were finally centrifuged (10 min, 8000 × *g*, 4 °C) and the pellets were frozen at −80 °C until further use. Extraction of total RNA from the bacteria and qRT PCR assay were performed as described in Saujet et al.^43^.

### Whole transcriptome sequencing and analysis

Transcriptome analysis for each condition was performed using 4 independent RNA preparations. Libraries were constructed using the Illumina Stranded Total RNA Prep Ligation with RiboZero Plus (Illumina, USA) kit. The ribodepletion step was carried using specific probes synthesized specifically to target *C. difficile* ribosomal sequences (Supplementary Table 1). After ribodepletion, libraries were prepared according to the supplier’s recommendations. RNA sequencing was performed on the Illumina NextSeq 2000 platform using 67 bases for a target of 10 M reads per sample.

### Electromobility shift assays (EMSA)

Only freshly purified CD1687 from *E. coli* were used in these assays. CD1687 (from 0.5 µM to 16 µM) was incubated with DNA (pUC9 or PCR product) in 10 μl of sodium phosphate buffer (50 mM; pH = 8.0) for 30 min at room temperature. Samples were loaded and migrated on TAE buffered agarose gels (1% w/v) for 90 min at 100 V. Controls were performed with CD1687 denatured at 100 °C for 15 min before the assay. Gels were stained with ethidium bromide and pictures were taken with an Amersham ImageQuant 800 (Cytiva). The pUC9 plasmid was prepared from *E. coli* stock using the Nucleospin plasmid kit (Macherey-Nagel, Germany) and the PCR amplicon used was generated using *C. difficile* 630*Δerm* as the DNA template and primers targeting the region of *CD1438* (Supplementary Table 1). gDNA was extracted from cell culture using the DNeasy Blood & Tissue Kit (QIAGEN, Netherlands).

### 5’RACE experiment

A 5’RACE was performed using the 5’ RACE System for Rapid Amplification of cDNA Ends, version 2.0 kit (Invitrogen, USA). Briefly, cDNA was generated by reverse transcription from total RNA extract followed by degradation of the RNA. dC-tailing was then performed with the cDNA and the resulting dC-tailed DNA was used as the template in PCR as described in the kit instructions. The PCR products were analyzed by agarose gel electrophoresis (1% agarose in TAE buffer). To identify the transcription start sites, PCR products were inserted into the pGEM-T easy vector kit as described by the manufacturer (Promega, USA). Insert were then PCR-amplified and the resulting PCR products were sequenced.

### Epifluorescence microscopy

For microscopy, 48 h biofilms were generated in 96-well plates (black, Greiner) as described above, washed and 50 μl of the polyclonal anti-CD1687 antibodies diluted in PBS (400 ng/mL) was then added to each well and incubated overnight at 4 °C. The wells were carefully washed twice with PBS followed by the addition of a solution containing DAPI (1/1000 dilution) and secondary antibodies (goat anti-rabbit conjugated with Texas Red; 1/5000 dilution; Invitrogen, cat: T-2767) in PBS. The plates were incubated at room temperature for 2 h. Wells were then carefully washed with PBS and 200 μl of fresh PBS was added for data acquisition. Images were taken with the Nikon Eclipse Ti inverted microscope (Nikon, Japan).

### Bacteria-DNA binding assay

A 433 bp amplicon modified at one end with a C6 amine and corresponding to the region of the *CD1438* gene was used to covalently coat a DNA-BIND Surface 96-well plate (Corning, USA) according to the manufacturer’s guidelines. Briefly, 100 µL of a 250 nM solution of amplicon prepared in the binding buffer (50 mM sodium phosphate buffer pH = 8.5; 1 mM EDTA) were placed in the wells and the plate was incubated overnight at 4 °C. Control wells were made using only the binding buffer. Then, the wells were washed three times with 200 µL of PBS and the plate was introduced in the anaerobic chamber. Exponential phase cultures of the *Δ1687pDIA6920* strain (Supplementary Table 1) grown in BHISG and appropriate antibiotics with or without the ATc inducer, were diluted to an OD(600 nm) of 0.5 and 200 µL of these bacterial suspensions were placed in the wells of DNA-coated plate. The plate was incubated anaerobically at 37 °C for 20 min and then washed twice with BHISG before adding 200 µL of BHISG containing 25 µg of DNAse I in each well. The plate was incubated anaerobically for 20 min at 37 °C and bacteria were counted from suspension on BHI agar plates. The PCR amplification to obtain the modified amplicon was performed with chromosomal DNA of the *C. difficile* 630*Δerm* with primers targeting the region of *CD1438* (Supplementary Table 1).

### Statistical analysis

The biofilm assays, bacteria-DNA binding assay, and RT-qPCR were analyzed using a one-way ANOVA test followed by either a Tukey’s multiple comparison test or a Dunnett’s multiple comparison test.

### Reporting summary

Further information on research design is available in the Nature Research Reporting Summary linked to this article.

## Supplementary information

Supplementary Material

Reporting Summary

Supplementary table 1

Supplementary table 3

Supplementary table 4

Supplementary table 5

## Data Availability

RNA-Seq data generated in this study are available in the NCBI-GEO with the accession number GSE218475. The mass spectrometry proteomics data have been deposited to the ProteomeXchange Consortium via the PRIDE partner repository with the dataset identifier PXD038282.
