# Supplementary material for: The cell wall lipoprotein CD1687 acts as a DNA binding protein during deoxycholate-induced biofilm formation in Clostridioides difficile
Source: NPJ Biofilms Microbiomes. 2023 May 11;9:24. doi: 10.1038/s41522-023-00393-5 (PMC10175255; doi:10.1038/s41522-023-00393-5)
Supplement: Supplementary file 1 — Supplementary Material [file 41522_2023_393_MOESM1_ESM.pdf]

Supplementary material

Emile Auria[1], Lise Hunault[2,3], Patrick England[4], Marc Monot[5], Juliana Pipoli Da Fonseca[5], Mariette Matondo[6], Magalie Duchateau[6], Yannick D.N. Tremblay[7] and Bruno Dupuy[1*]

[1] Institut Pasteur, Université Paris-Cité, UMR-CNRS 6047, Laboratoire Pathogenèse des Bactéries Anaérobies, F-75015 Paris, France
[2] Institut Pasteur, Université Paris-Cité, INSERM UMR1222, Unit of Antibodies in Therapy and Pathology, Paris, France
[3] Sorbonne Université, INSERM, CNRS, Centre d'Immunologie et des Maladies Infectieuses (CIMI-Paris), F-75013 Paris, France
[4] Plateforme de Biophysique Moléculaire, Institut Pasteur, CNRS UMR3528, Paris, France.
[5] Plateforme technologique Biomics, Institut Pasteur, Paris, France
[6] Plateforme Proteomic, Institut Pasteur, France
[7] Department of Biochemistry, Microbiology and Immunology, University of Saskatchewan: Saskatoon, SK, CA

* To whom correspondence should be addressed: BD: Institut Pasteur, Université de Paris cité, UMR-CNRS 6047, Laboratoire Pathogenèse des Bactéries Anaérobies, F-75015 Paris, France
Tel: 33140613175,
Email: bruno.dupuy@pasteur.fr

List of contents:

Supplementary Figure 1: Effect of DCA on the transcript levels of the *CD1685-CD1689* gene cluster
Supplementary Figure 2: Confirmation of gene deletion in the *CD1685-CD1689* cluster and biofilm formation by the resulting deletion strains.
Supplementary Figure 3: Analysis of the interactions between DCA and immobilized CD1687 by surface plasmon resonance.
Supplementary Figure 4: Functional classification of the differentially expressed genes identified in the transcriptomic analyses.
Supplementary Figure 5: Protein levels and localization of CD1687 on the surface of biofilm grown *C. difficile* cells by immunofluorescence.
Supplementary Figure 6: SDS-PAGE and Western immunoblot of proteins isolated from *E. coli* expressing CD1687 and Western immunoblot of CD1687 protein levels in different *C. difficile* strains.

Supplementary Table 1: Lists of strains, plasmids and primers used in this study
Supplementary Table 2: Sequencing results from the DNA amplicons generated by 5'RACE
Supplementary Table 3: Major transcriptomic changes observed in the transcriptomic experiments
Supplementary Table 4: List of common genes differently expressed in both transcriptomic experiments
Supplementary Table 5: Protein isolated by pull-down

<u>Supplementary figures</u>

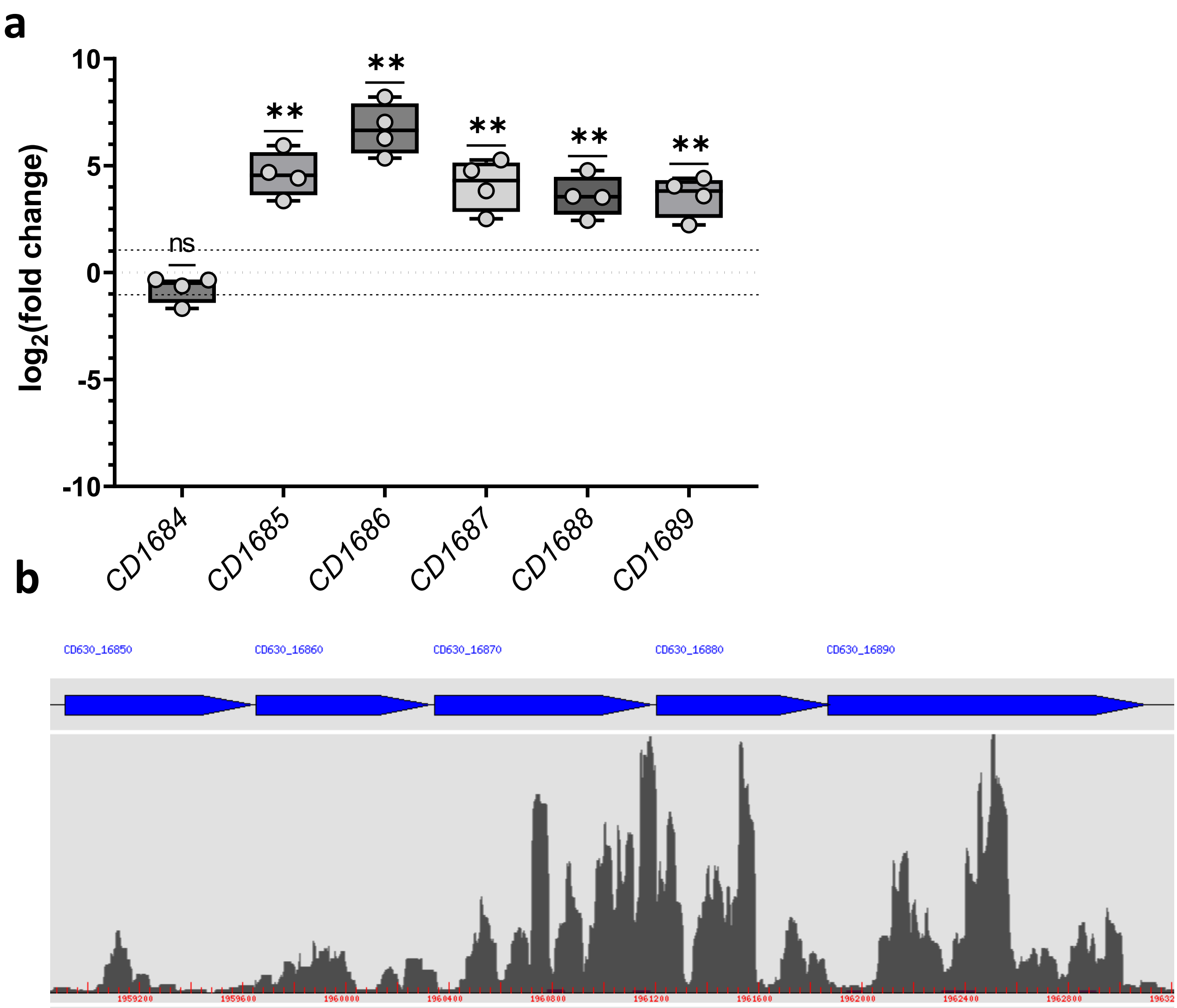

Supplementary Figure 1: Effect of DCA on the transcript levels of the *CD1685-CD1689* gene cluster. a. RNA was extracted from *C. difficile* strain 630*Δerm* grown for 48h in BHISG with or without DCA (240µM). Comparative RT-qPCR analysis of the *CD1685-CD1689* genes were performed between cell grown in the presence and in the absence of DCA. **b.** Raw read numbers detected along the *CD1685-CD1689* region provided by the transcriptome published in Dubois *et al.* (2019) from cells grown in BHISG with 240µM DCA for 48h. The boxplot used to represent quantitative data figure the median, minimum, maximum and upper and lower quartiles.

Asterisks indicate statistical significance with a t test comparing the theoretical mean of 0 (ns: not significant; **: p<0.01)

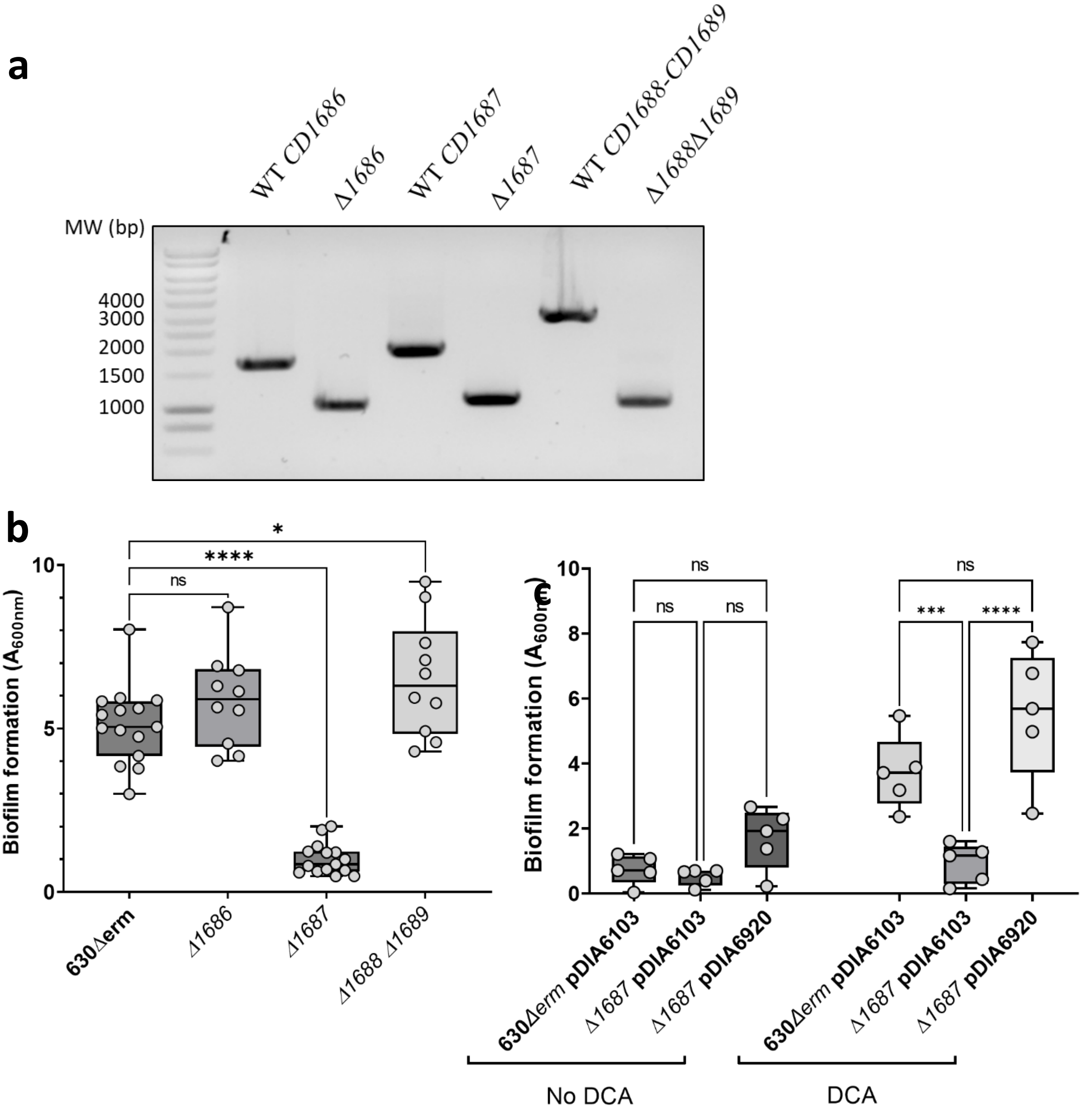

Supplementary Figure 2: Confirmation of gene deletion in the *CD1685-CD1689* cluster and biofilm formation by the resulting deletion strains. a PCR amplification using DNA from the wild type strain, the *Δ1686 strain*, *Δ1687* strain and the *Δ1688Δ1689* strain was performed using the "verification" primers listed in Table S1. In the WT/630*Δerm* strain, the expected sizes of the PCR products were 1625bp, 1820bp, and 2809bp for *CD1686*, *CD1687* and *CD1688-CD1689* genes, respectively*.* In the deletion strains, the expected sizes of the PCR products were 968bp, 995bp and 939bp for the *Δ1686, Δ1687,* and *Δ1688- Δ1689* deleted genes, respectively. **b.** Biofilms formation by the wild type (630*Δerm*), the *Δ1686,* the *Δ1687* and the *Δ1688Δ1689* strains was assayed 48h after inoculation in BHISG with DCA (240µM), **c.** Biofilms formation by wild type strain complemented with the control plasmid (pDIA6103) and the *Δ1687* mutant strain complemented with the *CD1687* plasmid (pDIA6920) or the control plasmid (pDIA6103) was assayed 48h after inoculation in BHISG with ATC (100ng/mL), in the presence or not of DCA (240µM). Each data point represents an independent biological replicate composed of 2 to 4 technical replicates. The boxplots used to represent quantitative data figure the median, minimum, maximum and upper and lower quartiles.

Asterisks indicate statistical significance with a one-way ANOVA test followed by a Tukey's multiple comparison test (ns: not significant; *: p<0.05; ***: p<0.001; ****: p<0.0001).

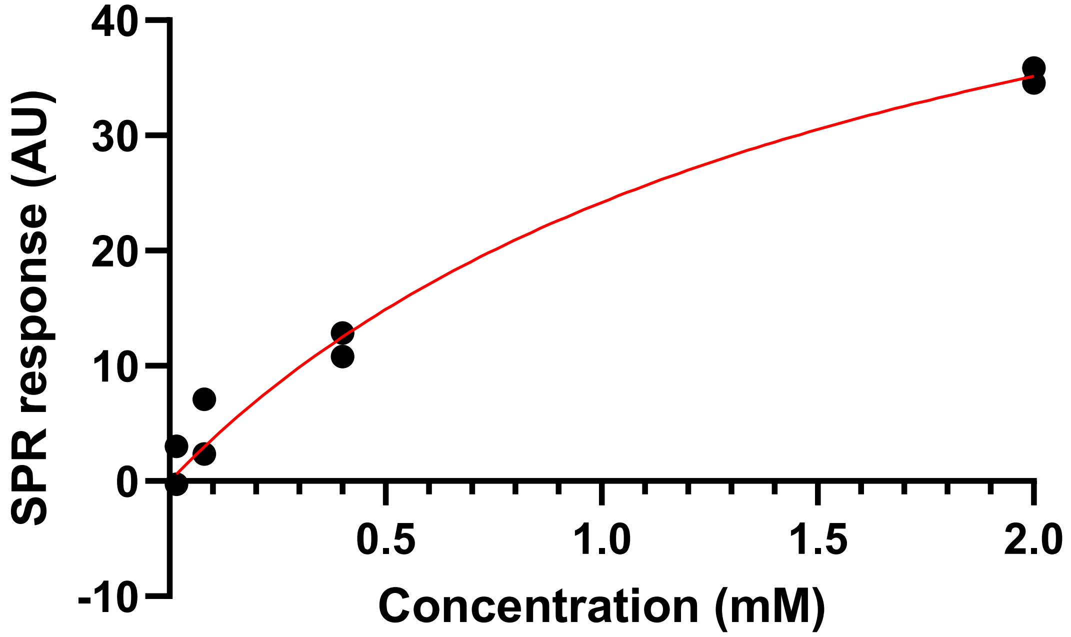

Supplementary Figure 3: Analysis of the interactions between DCA and immobilized CD1687 by surface plasmon resonance. The specific steady-state surface plasmon resonance responses were determined and plotted against the DCA concentration, allowing to determine the affinity and stoichiometry of the interaction between CD1687 and DCA. Dots represent the raw data while the red curve represents the fitted model. Here we determined that the dissociation constant of the DCA-CD1687 complex is 1.65±0.58mM and the stoichiometry is of 5±1 DCA molecule per CD1687 protein.

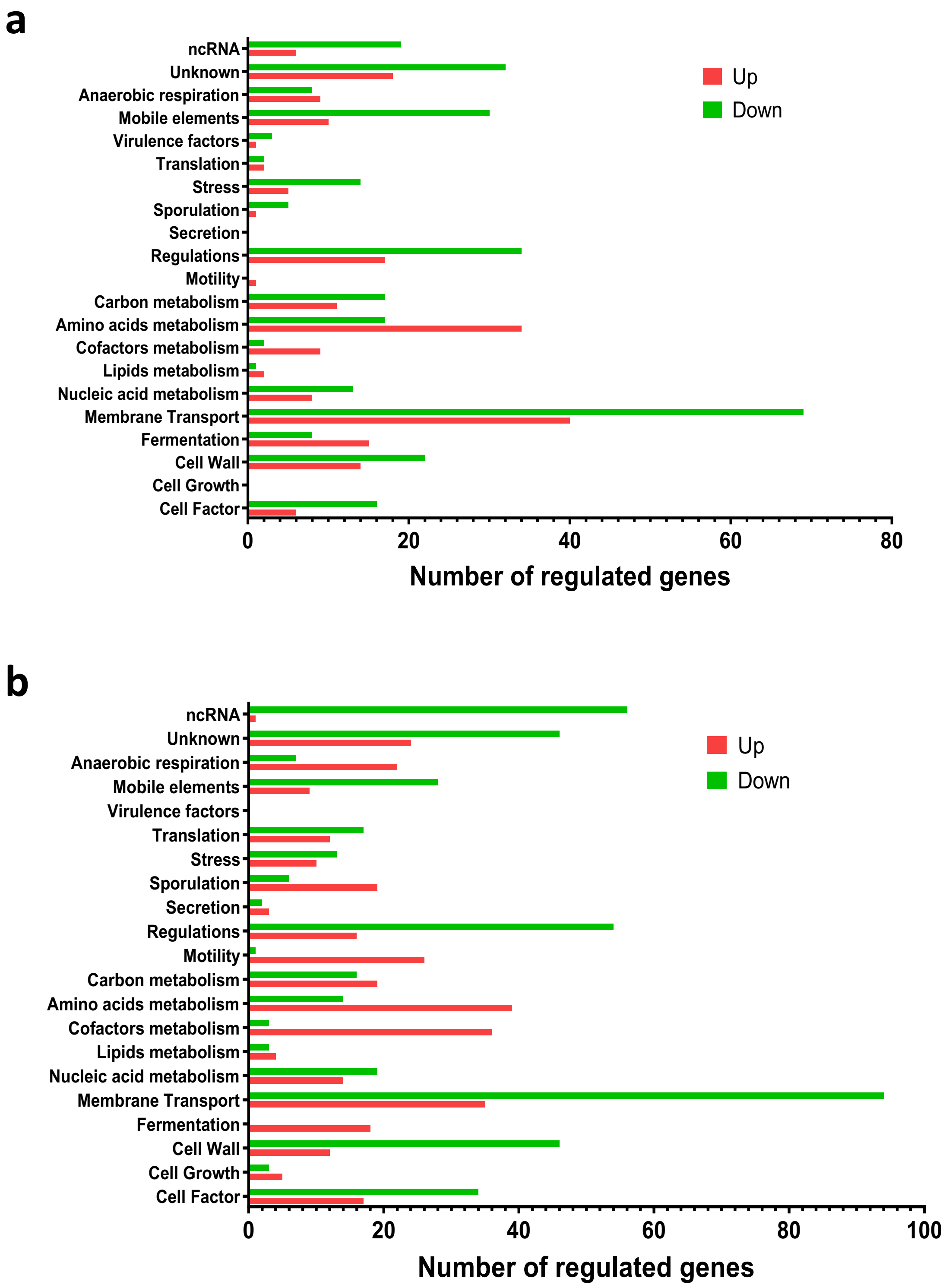

Supplementary Figure 4: **Functional classification of the differentially expressed genes identify in the transcriptomic analyses. a.** Transcriptome comparison between the wild type 630*Δerm* and the Δ*1687* mutant strain grown in BHISG in presence of DCA (240µM) for 24h. **b.** Transcriptome comparison between the wild type strain carrying an inducible plasmid overexpressing CD1687 (pDIA6920) grown in BHISG in the presence and absence of the ATC inducer for 24h. Changes in expression are colour coded (in red, up-regulated; or green, down-regulated). Genes were classified by functional classes and the numbers of genes are reported on the x-axis of the graphs.

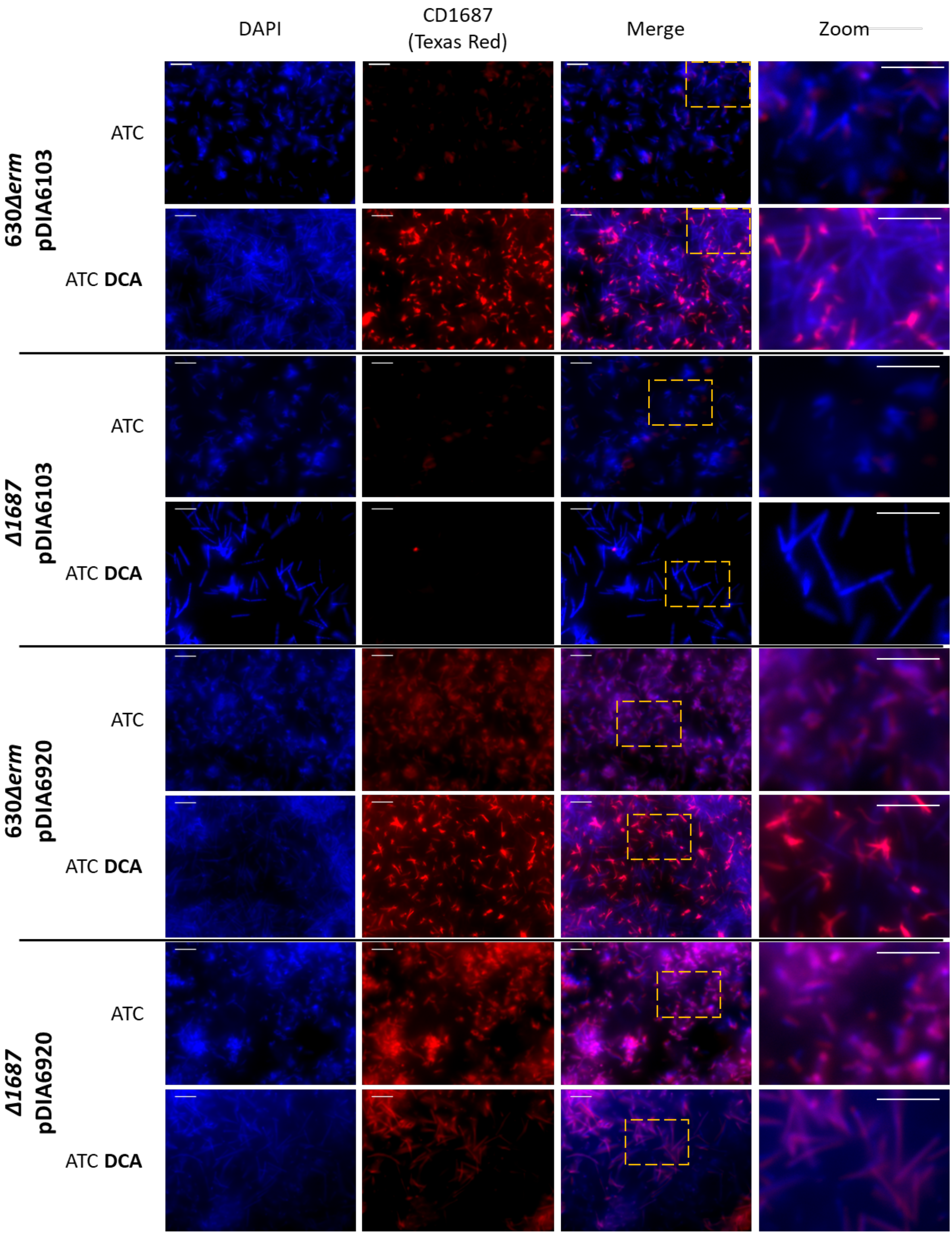

Supplementary Figure 5: Protein levels and localization of CD1687 at the cell surface of *C. difficile* cells within biofilm by immunofluorescence. *In situ* epifluorescence microscopy analysis was performed on the wild type strain (630*Δerm*) and the *Δ1687* mutant strain carrying the *CD1687* plasmid (pDIA6920) or the empty control plasmid (pDIA6103) grown as 48h biofilm in BHISG with ATC (100ng/mL), with or without DCA (240µM). DNA was stained with DAPI (blue) and CD1687 was detected with a specific anti-CD1687 rabbit antibody detected with a Texas Red-conjugated goat anti-rabbit antibody (red). Pictures are representative of three biological replicates and were taken with a Nikon Eclipse Ti inverted microscope (Nikon, Japan). Scale bar: 10µm.

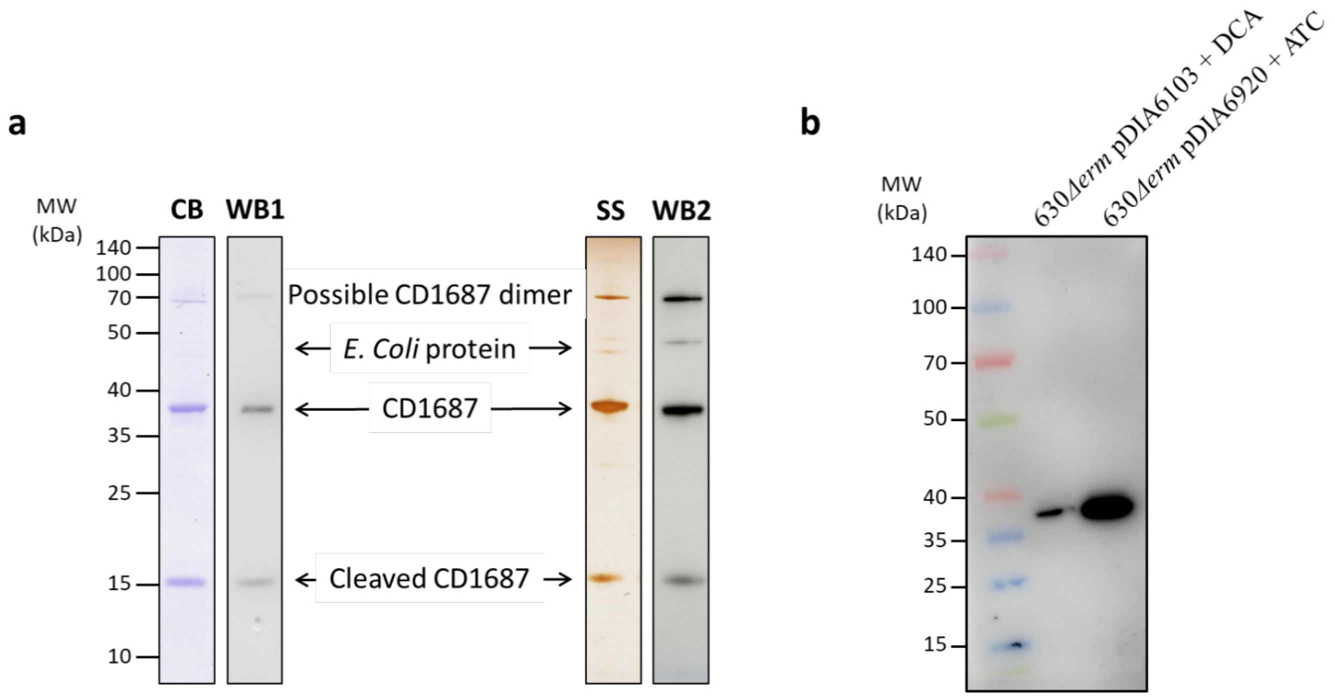

Supplementary Figure 6: SDS-PAGE and western immunoblot of the CD1687 protein expressed from *E. coli* or from different *C. difficile* strains. **a.** Analysis of the protein purified from *E. coli* expressing the CD1687-his$_6$ protein by silver staining (SS), Coomassie blue coloration (CB) and Western Blot (WB) using either a mouse anti-his$_6$ antibody (Invitrogen) (WB1) or an anti-CD1687 rabbit antibody (WB2). The CD1687 dimer and the CD1687 truncated protein form of CD1687 were detected only in *E. coli* extracts. **b.** CD1687 protein levels detected by western immunoblot using an anti-CD1687 antibody from protein extracted from the wild type with the control vector pDIA6103 or the complementation *CD1687* plasmid (pDIA6920). The bacteria were grown for 48h in BHISG supplemented either with ATC (100ng/mL) or DCA (240µM). 2µg of total proteins were loaded in each running lane.

<u>**Supplementary Table**</u>

Supplementary Table 2: Sequencing results from the DNA amplicons generated by 5'RACE

	Last sequenced 30bp	TSS	50bp upstream
1	GAATATATGATGTCTATACAAGATATAAAA	TSS1	TGATGTTTGATAGGGGAAAATGGAATAAATTGGATTATAATGAACTTTTA
2	GGAAATTGGAAGGAATTTTTAGAAGTAGCA	TSS1	TAGGTATTAGAGTTCCTAATTTAAGAAAGTTATCAAAAGAAATAAGCCAA
3	ACATATTAAAGAAATATTTGAATACTGTAA	TSS2	GAAATTAGATTTGCATTAGTTATGTTTTTAATATATTATGTTGATGATAA
4	ACATATTAAAAGACGAATTAAATTATAATA	TSS3	TTTAAGAACTTTTTTAAATAAAAAAGGATATAAGTCAGAAAAAGGTAAAT
5	CATATTAAAAGACGAATTAAATTATAATAA	TSS3	TTAAGAACTTTTTTAAATAAAAAAGGATATAAGTCAGAAAAAGGTAAATA
6	ATTAAATTATAATAAAAGTTTGGATAATAG	TSS3	AATAAAAAAGGATATAAGTCAGAAAAAGGTAAATACATATTAAAAGACGA
7	ACATATAGATGATGAACACATAGATGAATT	TSSa	GTAAAAGATTACAATATAAAGCCTAAAAAAATATCTAATAAAAAAGATGT
8	ACACATAGATGAATTAAAAACAACAAGAAT	TSSa	ATAAAGCCTAAAAAAATATCTAATAAAAAAGATGTACATATAGATGATGA
9	TATAAAAATCTACTATAAAATACTACTATA	TSSb	TTGAAAAACATCAAATACTTACACAGGCATTGAAAGAATTTATGAAAAAG

red: putative -10 box ; green: putative -35 box
